# Gene Expression and Photophysiological Changes in *Pocillopora acuta* Coral Holobiont Following Heat Stress and Recovery

**DOI:** 10.3390/microorganisms8081227

**Published:** 2020-08-12

**Authors:** Rosa Celia Poquita-Du, Yi Le Goh, Danwei Huang, Loke Ming Chou, Peter A. Todd

**Affiliations:** 1 Experimental Marine Ecology Laboratory, S3 Level 2, Department of Biological Sciences, National University of Singapore, 16 Science Drive 4, Singapore 117558, Singapore; dbsgyl@nus.edu.sg (Y.L.G.); dbspat@nus.edu.sg (P.A.T.); 2 Tropical Marine Science Institute, National University of Singapore, S2S Building 18 Kent Ridge Road, Singapore 119227, Singapore; huangdanwei@nus.edu.sg (D.H.); tmsclm@nus.edu.sg (L.M.C.); 3 Reef Ecology Laboratory, S3 Level 4, Department of Biological Sciences, National University of Singapore, 16 Science Drive 4, Singapore 117558, Singapore

**Keywords:** adaptive, immune response, heat, reversible phenotypic response, RT-qPCR

## Abstract

The ability of corals to withstand changes in their surroundings is a critical survival mechanism for coping with environmental stress. While many studies have examined responses of the coral holobiont to stressful conditions, its capacity to reverse responses and recover when the stressor is removed is not well-understood. In this study, we investigated among-colony responses of *Pocillopora acuta* from two sites with differing distance to the mainland (Kusu (closer to the mainland) and Raffles Lighthouse (further from the mainland)) to heat stress through differential expression analysis of target genes and quantification of photophysiological metrics. We then examined how these attributes were regulated after the stressor was removed to assess the recovery potential of *P. acuta*. The fragments that were subjected to heat stress (2 °C above ambient levels) generally exhibited significant reduction in their endosymbiont densities, but the extent of recovery following stress removal varied depending on natal site and colony. There were minimal changes in chl *a* concentration and maximum quantum yield (Fv/Fm, the proportion of variable fluorescence (Fv) to maximum fluorescence (Fm)) in heat-stressed corals, suggesting that the algal endosymbionts’ Photosystem II was not severely compromised. Significant changes in gene expression levels of selected genes of interest (GOI) were observed following heat exposure and stress removal among sites and colonies, including *Actin*, calcium/calmodulin-dependent protein kinase type IV (*Camk4*), kinesin-like protein (*KIF9*), and small heat shock protein 16.1 (*Hsp16.1*). The most responsive GOIs were *Actin*, a major component of the cytoskeleton, and the adaptive immune-related *Camk4* which both showed significant reduction following heat exposure and subsequent upregulation during the recovery phase. Our findings clearly demonstrate specific responses of *P. acuta* in both photophysiological attributes and gene expression levels, suggesting differential capacity of *P. acuta* corals to tolerate heat stress depending on the colony, so that certain colonies may be more resilient than others.

## 1. Introduction

Coral reefs are faced with severe degradation due to anthropogenic activities and the progression of climate change is exacerbating these stresses even further [1]. Perhaps the most apparent manifestation of global warming is an increase in sea surface temperature [2], affecting coral reefs worldwide [3,4]. This temperature rise can pose a threat not only to corals but also to a wide range of marine organisms [5]. Previous observations have shown impacts of elevated temperature on various biological processes crucial for coral survival such as calcification, immune and stress responses, as well as reproductive capacity [6,7]. When corals are exposed to heat beyond their tolerance limits, bleaching can occur such that corals dissociate from their photosynthetic endosymbionts which can be observed physically by the loss of pigmentation [8,9]. Severe bleaching of corals can lead to physiological damage, mass mortality, and changes in reef community composition [10,11,12].

The ability to constantly sense and exhibit flexibility to environmental changes is important for all organisms to maintain cellular homeostasis [13]. It is especially critical for corals as they are sessile and therefore more vulnerable to environmental variation than motile animals. Corals are known to exhibit a range of mechanisms to cope with changes in environmental conditions [14], including phenotypically plastic short-term reversible responses [15]. Phenotypic plasticity is defined as the capacity of an organism to alter a specific aspect of its phenotype, within its lifetime, in response to changes in its environment [16,17,18]. There are usually fitness costs and limitations associated with plasticity, and the degree of plastic responses exhibited depends on a balance between these factors and the overall benefits to the organism. Acclimatization is an example of plastic response that increases fitness in order to face environmental change [19].

Acclimatization mechanisms in corals generally include orchestration in levels of gene and protein expressions [20,21], heterotrophic activities [22], physiological attributes [23], and skeletal modifications [24,25]. More recently, studies on coral acclimatization mechanisms have focused on differential gene expression analysis as alteration in gene expression profiles is regarded as the underlying mechanism for phenotypic plasticity [26]. Gene expression links genotype to phenotype and plays a central role in cellular adaptation to environmental changes [27]. While gene expression analysis has emerged as a powerful tool in assessing acclimatization capacity of a coral species, one of the current challenges is the variability in gene expression levels among and even within coral colonies [28,29,30,31]. Different degrees of acclimatization capacity among coral colonies are generally expected [16,32] and are mostly influenced by genotype and natal environment, where the colony is from [33]. For example, in a reciprocal transplantation experiment of *Porites astreoides* from different populations—inshore versus offshore—a positive correlation between cellular stress response genes and measurement of fitness (i.e., growth, energetic stores, endosymbiont density, and chlorophyll content) was found for inshore coral fragments, suggesting enhanced plasticity is beneficial for this population of corals [34]. However, a negative correlation between the above-mentioned attributes was found for offshore coral fragments implying negative trade-offs (‘costs’) of the plastic response [34].

During a more recent coral bleaching events in Singapore, the coral genus *Pocillopora* appeared to show a shift to being less susceptible [12,35], similar to observationsfrom the Great Barrier Reef, Australia [36,37]. While this genus is known to be highly plastic [24,38,39], the mechanisms underlying its enhanced resistance to and ability to recover from thermal stress remain unclear. Therefore, it is of interest to explore both photophysiological and transcriptional plasticity in *Pocillopora*, using *Pocillopora acuta* as the model species. Here, we examine (1) how gene expression and photophysiological attributes (maximum quantum yield of Photosystem II (PII) (Fv/Fm, the proportion of variable fluorescence (Fv) to maximum fluorescence (Fm)), endosymbiont density, and chlorophyll (chl) *a* concentration) among colonies of *P. acuta* corals are regulated in response to heat stress; (2) how these responses are then moderated after the stressor is removed, to assess their capacities to recover; and (3) whether responses vary depending on coral natal site and colony.

## 2. Materials and Methods

### 2.1. Experimental Design

Six *P. acuta* colonies were collected from two sites—three colonies (labelled G1, G2, G3) were collected from a site near to Singapore’s mainland (Kusu, 1.2257° N 103.8602° E) and another three (G4, G5, G6) from further offshore (Raffles Lighthouse, 1.1602° N 103.7403° E). The colonies were collected at least ~10 m apart. Each colony was fragmented (total of 96 fragments = six colonies × two treatments × two periods × four replicates) and placed in a flow-through aquarium facility for 5 d of acclimation.

**Figure 1:**
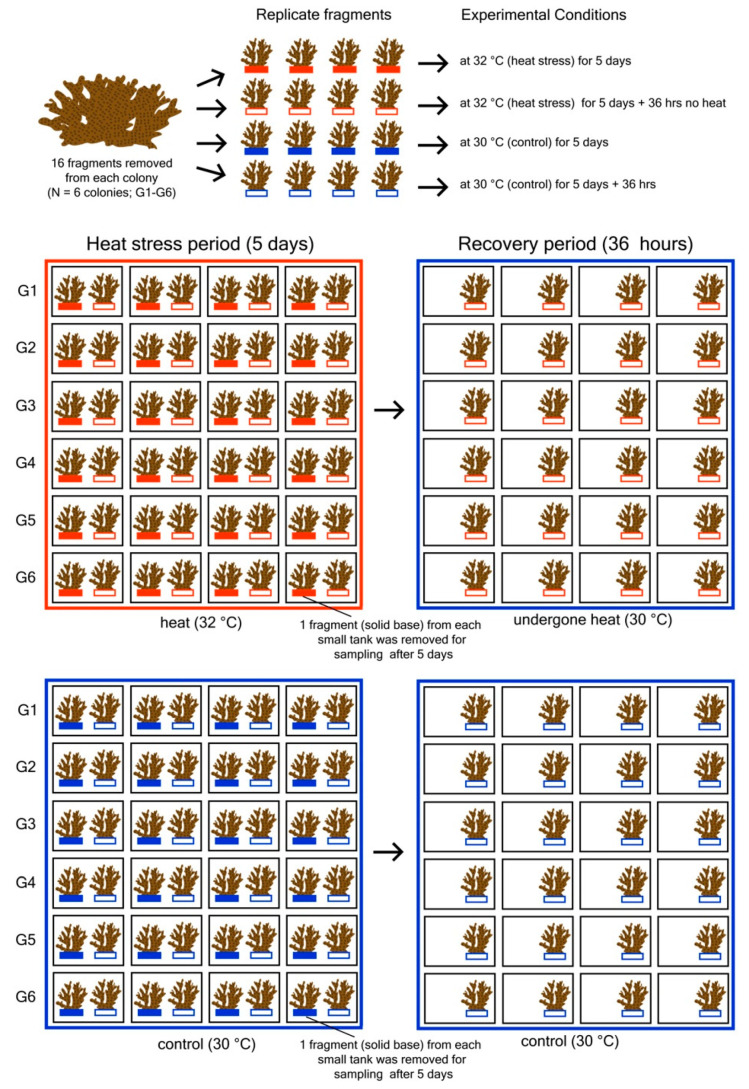
Experimental design showing each colony divided into fragments and assigned to two treatments (red fragment = heat, blue fragments = control) and periods (heat stress period, recovery period), totaling 96 fragments (two treatments × two periods × six colonies × four replicate fragments). A total of 24 small plastic tanks exposed to the heat treatment and 24 were controls; each contained two coral fragments from the same colony. Collection of 48 fragments (one from each small tank) was performed after 5 days for (destructive) analyses of endosymbiont density, chl *a* concentration, and gene expression levels. The remaining 48 fragments remained in their tanks, allowing the previously-heat stressed fragments to recover for 36 h at ambient temperature (30 °C). All 48 fragments were collected after the recovery period for analysis.

A common garden experiment was conducted to examine photo-physiological performances and gene expression levels of *P. acuta* fragments from six colonies under two temperature treatments: heat stress (32 °C) and ambient seawater temperature i.e., control (30 °C) at two periods (heat stress and recovery periods) (Figure 1). The “heat stress” period was created through repeated 4 h (10 a.m. to 2 p.m.) daily exposure of corals to heat (32 °C) for 5 d, while for the “recovery” period, corals were allowed to recover for 36 h at 30 °C. The assigned temperatures for both control and treatment were based on previous data for sea surface temperature (SST) in Singapore where maximum monthly mean was 29.86 °C and can rise ≥1 °C above this level during warmer periods (around April to July) [12,35,40]. The experiment was carried out in early July 2017, which falls within the warmer months, however, during this period, SST peaked at only 30.7 °C in May and there were no reports of coral bleaching in local reefs [41].

Small rectangular plastic tanks (48 in total) were gravity-supplied with seawater from reservoirs and provided with constant aeration. All the small tanks were immersed in large fiberglass water baths maintained at two temperature levels: (1) 30 °C for the “control” and, (2) 32 °C for the “heat” treatment, using aquarium heaters (EHEIM Jagger). To ensure uniform temperature, a pump was used to circulate water throughout the bath. In each tank and water bath, temperature and light loggers (HOBO^®^U22-001 and UA-002-08, Onset Computer Corporation, Bourne, MA, USA) were set to log continuously every 5 min for the duration of the experiment. The tanks within the water bath were rearranged every 2 days to avoid any positional effects. Two coral fragments from the same colony were placed in each small tank. One fragment from each was collected after 5 days (the heat stress period) and used for destructive procedures, including sampling chl *a* concentration, endosymbiont density, and RNA extraction. The second fragment was collected after an additional 36 h for post-recovery sampling of the same photophysiological parameters mentioned above and gene expression levels.

### 2.2. Quantification of Maximum Quantum Yield (Fv/Fm), Endosymbiont Density, and Chlorophyll (chl) a Concentration

Measurements of Fv/Fm, chl *a* concentration and endosymbiont density were taken from all coral fragments before the start of experiment, following “heat stress” (48 samples) and “recovery” (48 samples) periods. Measurements of Fv/Fm were conducted using a portable diving pulse amplitude modulating (PAM) fluorometer (Heinz Walz GmbH, Effeltrich, Germany) before sunrise, from 0300 to 0700, to avoid any influences from changes in natural ambient light. Each fragment was sampled five times, aiming at different polyps on ends of upward-pointing branches and keeping a fixed distance of 5 mm between the fiber optic sensor and the coral surface [42]. All readings for each fragment sample were averaged.

Immediately after the heat stress period, 48 coral fragments (six colonies × two treatments × four replicates) were collected, wrapped in foil, and transferred to a cooler box for subsequent endosymbionts extraction following procedures from Ben-Haim et al. (2003) [43]. Another 48 coral samples were collected (from the coral fragments designated for the “recovery” period) 36 h after the heat stress was removed. To remove coral tissue from the skeleton, a Waterpik^®^ water flosser was used and filled with filtered seawater. Mucus and clumping of endosymbiont cells were minimized by filtering the resultant slurry through 50 and 15 µm plankton mesh nets. Cells were pelleted from the slurry by centrifugation (Eppendorf 5810R) of 4000 rpm for 30 min at 20 °C. Subsequently, the pellets were resuspended in 5 mL filtered seawater and stored at −20 °C until further analysis.

All samples containing endosymbiont suspension were vortexed to mix. Aliquots of 1 mL from the 5 mL suspension were set aside for cell counting and the remaining 4 mL saved for chl *a* extraction. From the 1 mL aliquot, a subsample of 10 µL was taken out for enumeration of endosymbiont cells using a Neubauer improved hemocytometer under a compound microscope, performed eight times. Prior to cell counting, aliquots were passed through a 27G × ½ inch syringe to minimize clumping of endosymbiont cells. Subsequently, chl *a* was extracted from the endosymbionts pelleted from the 4 mL aliquot using 100% acetone for 24 h at 4 °C. Pigment absorbance readings were taken at 630, 663, and 750 nm using a UV-Vis spectrophotometer (UV-1280, Shimadzu, Japan) and chl *a* concentration was calculated using the equations from Jeffrey and Humphrey (1975) [44].

All fragments were photographed at a fixed vertical position with a ruler for image scaling. Using ImageJ (v. 1.50i), the boundary of a fragment from the background was delimited by tracing the shape of the fragment using both the polygon and wand tools and surface area was subsequently calculated using the integrated ‘measure’ function. As *P. acuta* is a branching coral, two opposite sides of the fragment were photographed to obtain a more representative surface area measurement. Finally, average values for endosymbiont cell density and chl *a* concentration from each sample were normalized against coral surface area.

### 2.3. RNA Extraction and Reverse Transcription (RT)

In conjunction with coral sample collection for both periods described above, coral nubbins (≈2 cm branches) were removed from the replicate fragments for both treatments and placed in individual sample tubes pre-filled with RNAlater (ThermoFisher Scientific, Singapore) for stabilization. The sample tubes were inverted to mix for 30 s and kept at 4 °C overnight to allow complete penetration of RNAlater into the coral tissues. The tubes were subsequently stored at −80 °C until RNA extraction. Total RNA isolation was performed using TRIzol (Life Technologies, Sigma-Aldrich, Singapore), following manufacturer’s protocol with slight modification to the homogenization procedure based on Barshis et al. (2013) [20]. RNA quality was checked by examining with gel electrophoresis for presence of clear bands of ribosomal RNAs, and RNA concentration was estimated using Qubit (RNA Broad Range Assay Kit, ThermoFisher Scientific, Singapore).

For each sample, complementary DNA (cDNA) was immediately prepared from the amount of total RNA equivalent to 1 µg, using a one-tube format of Bio-Rad iScript RT supermix for RT-qPCR (reverse transcription quantitative polymerase chain reaction). Reaction setup was composed of iScript RT supermix (4 µL), RNA template (varied depending on RNA sample concentration; 14.6–200 ng/µL), and nuclease-free water (variable), with final volume of 20 µL. Incubation of reaction mix was performed in a Labcycler (Sensoquest, Göttingen, Germany) following the manufacturer’s protocol: priming for 5 min at 25 °C, RT for 20 min at 46 °C, and RT inactivation for 1 min at 95 °C.

### 2.4. Primer Design and Validation

The selected genes of interest (GOIs) and internal control genes (ICGs) were derived from an RNA-Seq experiment conducted on *P. acuta* following heat exposure [38] as being the most responsive and stable, respectively. The transcript sequences used for primer designs were validated to be associated with corals only as reported in Poquita-Du et al. [38]. The full list of candidate genes is shown in Table 1.

Transcript sequences from Poquita-Du et al. [38] that matched selected GOIs were consolidated for alignment by gene for primer design. Primers for each gene were designed using the online tool by NCBI that incorporated Primer3 and BLAST (https://www.ncbi.nlm.nih.gov/tools/primer-blast/, June 2017), specifying “*Pocillopora acuta*” in the target organism field. Primer parameters include GC content = 50–60%; Tm (melting temperature) = Min 58 °C, Max 60 °C, Tm difference = 1 °C; PCR product size = Min 100, Max 1000. Forward primer sequences for each gene were searched in the consensus gene sequence used for the primer design. The reverse primer sequences were reversed using an online reverse primer tool (https://www.bioinformatics.org/sms/rev_comp.html, June 2017) and similarly searched against the consensus sequences.

The specificity of each primer pair for each GOI was verified by PCR using the GoTaq Green Master Mix. PCR reaction steps were (1) denaturation: 95 °C for 45 s (2) annealing: start at ~55 °C, increasing in increments of 1 °C to annealing temperature (60 °C) for 45 s; (3) extension: 72–74 °C for 5 min, repeated for 30 cycles. Gel electrophoresis was performed to check for successful amplification of target regions. Presence of gDNA contamination was also assessed using a no-RT control.

Efficiencies of primers were determined by amplifying a series of dilutions of *P. acuta* cDNA covering two orders of magnitude of template amount (0.078–5 ng) using qPCR (CFX96, Bio-Rad Laboratories, Hercules, California, USA). Calculations of efficiencies (E, the amplification factor per PCR cycle) needed to correct for amplification efficiencies per primer were done using MCMC.qpcr package in R developed by Matz et al. (2013) [45]. The function, PrimEff( ), calculates E and plots the regression slopes and E based on dilution series. The GOIs with E values outside the 1.85–2.15 range had primers redesigned and re-validated. GOIs which failed amplification were excluded from downstream analyses.

### 2.5. Quantification of Gene Expressions

RT-qPCRs (15 µL volume per reaction) were performed in Bio-Rad CFX96 using SsoAdvanced inhibitor-tolerant SYBR Green Supermix following the manufacturer’s protocol (polymerase activation and DNA denaturation: 3 min at 98 °C, denaturation: 98 °C for 15 s, annealing/extension: 60 °C for 45 s, and plate read) repeated for 40 cycles. For each RT-qPCR run, three wells (technical replicates) were prepared for each cDNA sample in a 96-well hard-shell low-profile PCR plates with two wells each for no template and no-RT controls. Melting curve analysis (65–95 °C, increment of 0.5 °C every 5 s, and plate read) of the amplification product obtained was performed to further validate specificity of each primer. To control for variations in expressions of genes due to differences in RNA concentration of each sample, amount of cDNA template was standardized to ~10 ng of cDNA for every reaction mix.

### 2.6. Statistical Analyses

To examine the effects of “colony”, “site”, “treatment”, and “period” on coral photophysiological performances, data were fitted with linear mixed models for Fv/Fm and chl *a* concentration (continuous data) and generalized linear mixed model for endosymbiont density (count data) using lme4 package in R [46]. The fixed effect “colony” was nested within “site” to examine whether responses of corals within a site vary depending on the colony. The term “period” here does not refer to different time points of a treatment, but, rather, the effect of ‘heat stress removal’ for testing whether there were changes in the response of corals which had been subjected to heat, relative to the control samples (i.e., control fragments allotted mainly for the recovery period). The models for all photophysiological metrics contained “sample replicate” as a random effect. Stepwise model simplification and selection were based on Akaike information criterion. Visual inspections of the residual plots were performed to ensure assumptions of normality and homoscedasticity were met for all models.

Data obtained from RT-qPCRs expressed as “cycle of quantification values” (i.e., Cq values) were collated and sorted for subsequent analysis. The data contained raw Cq values from all biological (*n* = 96 samples from six colonies × two treatments × two periods × four replicates) and technical (three RT-qPCR reactions per sample) replicates for each GOI. Following the Bayesian methodology outlined by Matz et al. (2013) [45], the Cq values were converted into molecule counts with corrections for primer efficiencies using the MCMC.qpcr package in R. This approach relies on calculated amplification efficiencies (E) per gene and estimate of Cq for a single target molecule using a formula: Count = E(Cq1-Cq). The Cq-to-counts conversion is the key transformation in this method in which higher variation at the low gene expression values is properly accounted for by the relative quantification model. The transformation makes it possible to fit the resulting data to generalized linear mixed models to account for Poisson-distributed fluctuations when the number of molecule count is low. Similar Bayesian approaches for analyzing qPCR data have been used in several other reports [47,48,49,50,51]. While *Actin* was designated as an ICG, it was found to be differentially expressed following heat stress and, therefore, no longer utilized as an internal control here and the model was run in the “naive” form (i.e., no control gene). While specification of control genes can sharpen estimates of model parameters, this was not critical as all normalizations can be performed within the model [45].

Each gene profile was examined to determine (1) treatment responses, (2) whether changes of gene expressions for corals previously exposed to heat occurred following the recovery period, (3) among-site variation in responses, and (4) influence of colony. The model contained fixed effects: “treatment”, “period”, “site”, and “colony”; a global fixed effect: “RNA quantity” (14.6–200 ng/µL) and a random effect: “sample replicate” that denotes individual cDNA preparations to account for unequal cDNA template loading between samples as a form of normalization.

## 3. Results

### 3.1. Photophysiological Performance

**Figure 2:**
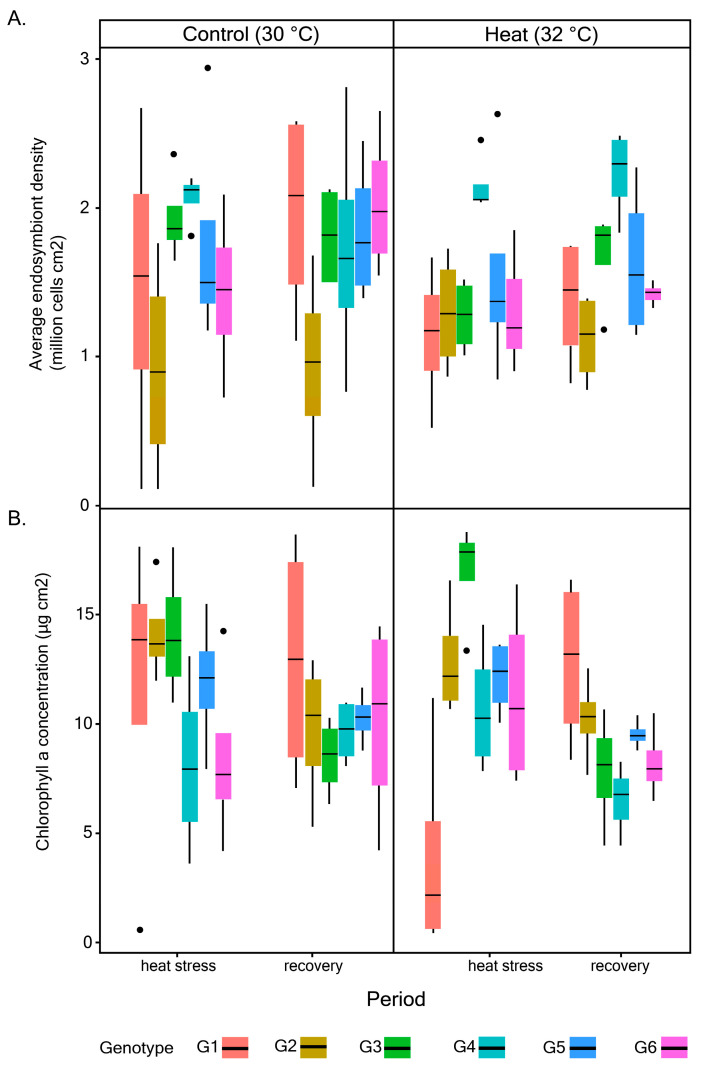
Changes in average endosymbiont density (**A**) and chl *a* concentration (**B**) for all colonies across experimental conditions.

Endosymbiont densities of *P. acuta* corals were significantly influenced by treatment (Chisq = 3.14 × 10^5^; df = 1; *p* = 2.2 × 10^−16^), period (Chisq = 2.07 × 10^5^; df = 1; *p* = 2.2 × 10^−16^), site (Chisq = 7.33; df = 1; *p* = 0.007), and the interactions between these factors (Figure 2A; Table 2). However, the observed changes due to these factors significantly depended on the colony (Chisq = 2.58 × 10^6^; df = 16; *p* = 2.2 × 10^−16^).

**Figure 3:**
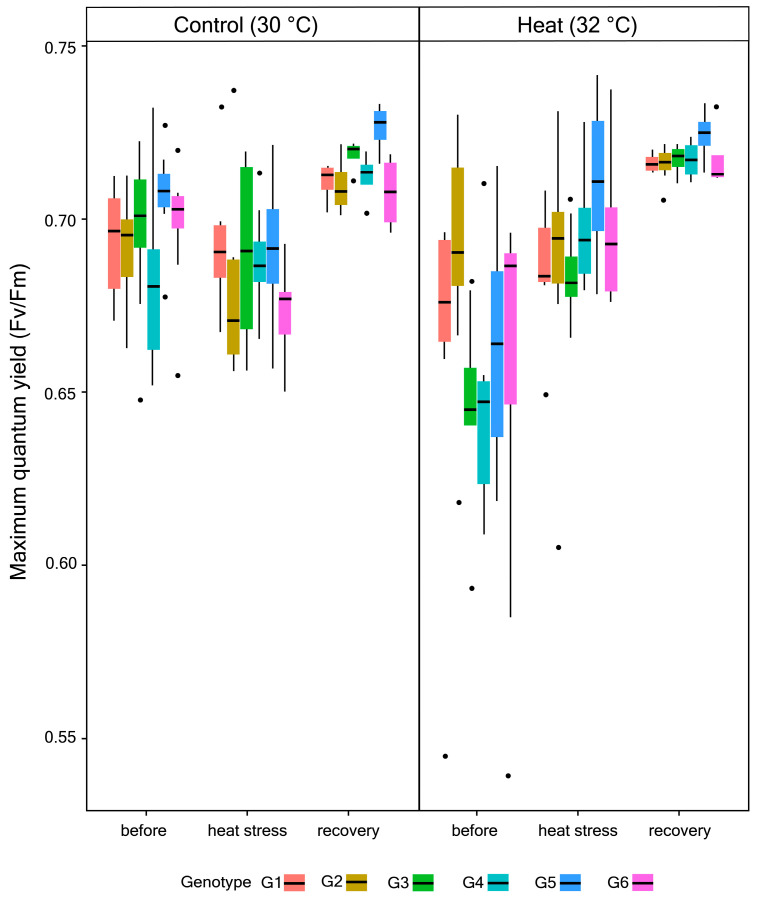
Changes in average Fv/Fm for all colonies across experimental conditions.

There were no significant changes for average chl *a* concentration in response to heat across periods and sites. However, there were significant differences in response among colonies and sites (Chisq = 41.572; df = 16; *p* = 0.0004) (Figure 2B; Table 3). While the main effect of heat on the average Fv/Fm of *P. acuta* corals was not significant, changes in Fv/Fm differed significantly between experimental periods (Chisq = 5.7531; df = 1; *p* = 0.016) (Figure 3; Table 4). There were no significant differences between sites and among colonies.

### 3.2. Primer Validation and Gene Expression Profiles

Among the nine candidate genes, only five (*Actin*, *Camk4*, *GAS8*, *Hsp16.1*, and *KIF9*) showed reliable amplification. Efficiency of amplification was high, within the range of acceptable values of 1.49–2.1 (see Kenkel et al. (2011)) [28] (Figure S1).

**Figure 4:**
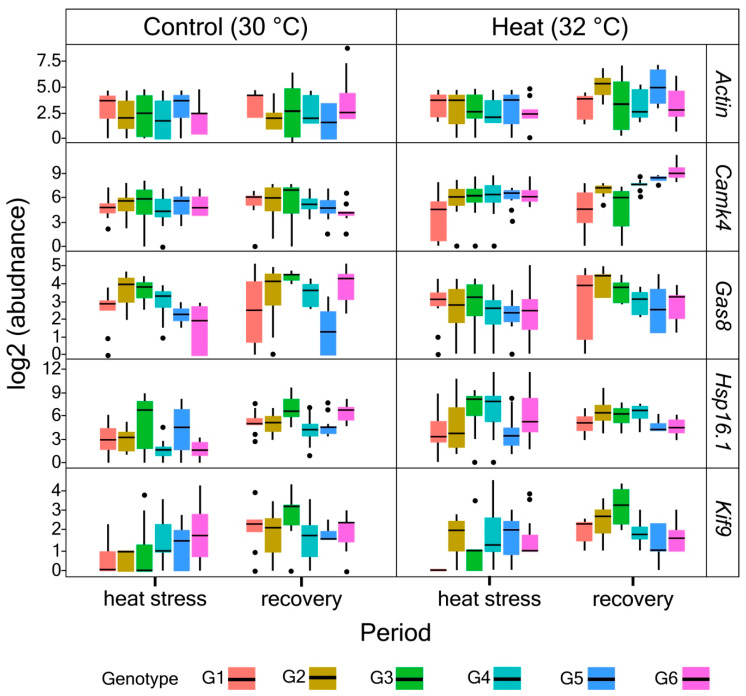
Changes in expression levels of all genes (log2-transformed) for all colonies examined across experimental conditions.

The main effect of heat on expression levels of all genes examined was significant for only *Actin* (post. (posterior) mean = −2.035; pMCMC < 0.001) (Figure 4 and Data S1). Following the recovery period, heat-stressed corals particularly from G2 showed significant increase in expression levels of *Actin* (post. mean = 2.41, pMCMC = 0.020). While the overall changes in expression levels of *Hsp16.1* and *KIF9* following heat exposure were not significant, there were colony-specific differences, particularly for G2 (post. mean = −2.10, pMCMC = 0.046; post. mean = 11.67m pMCMC = 0.038, respectively). Further, corals which were previously exposed to heat showed upregulation of *Camk4* during the recovery period, but this change was colony-specific with significant changes for G2 (post. mean = 4.28; pMCMC = 0.042) and G3 (post. mean = 8.02; pMCMC < 0.001). Expression levels of *GAS8* did not show any significant changes following heat stress and recovery among colonies and between sites.

## 4. Discussion

Our experiment tested how different colonies of *P. acuta* corals from two sites with differing distances to the mainland (Kusu (closer to the mainland) and Raffles Lighthouse (further from the mainland)) responded to heat stress and recover from it. Results showed that water temperature affected the photophysiological performances of *P. acuta* corals, after both the heat stress and recovery periods. Heat-induced differences in endosymbiont density between sites and among colonies were significant, as were changes in average chl *a* concentration. Both treatment and experimental period influenced F_v_/F_m_ and responses were similar across sites and colonies. Significant changes in expression levels of selected GOIs were also detected, however, the changes were mainly colony-specific for most GOIs examined except for *GAS8*. The most responsive GOIs were *Actin*, a major component of the cytoskeleton and the adaptive immune-related gene *Camk4*. Both genes exhibited downregulation following exposure to heat and colony-specific changes in heat-stressed corals upon removal of the stressor. Findings here underscore the capacity of *P. acuta* corals to achieve homeostasis under sub-optimal conditions and recover from perturbations by a combination of photophysiological regulation and orchestration of gene expression.

Coral endosymbionts are known to adjust their photophysiological attributes to suit the surrounding conditions. Rising water temperature can cause dissociation of endosymbiotic dinoflagellates from their coral host and lead to loss of pigmentation, from either reduction in endosymbiont density or chl *a* concentration [52,53]. However, the tendency of corals to undergo bleaching depends largely on the magnitude and duration of stress. Here, corals that were subjected to short-term heat stress (2 °C above normal levels) exhibited significant changes in their endosymbiont densities with specific responses dependent on colony and the natal site. Heat caused significant reductions in endosymbiont densities for all coral colonies, but the extent was less pronounced for corals from Raffles Lighthouse. However, one colony from this site (G4) showed significantly higher levels of endosymbiont density following heat exposure. A similar response has been observed previously for *Pocillopora* colonies exposed to heat stress (see [38,54]). Indeed, corals are known to regulate their endosymbiont abundance upwards under sub-optimal conditions to sustain high photosynthetic yield [55].

There were subsequent changes in endosymbiont densities for the heat-stressed corals after the stressor was removed but, again, there was variation in their recovery responses depending on colony and natal site. While there were significant changes in endosymbiont densities for most coral colonies, none of them showed visible changes in terms of tissue pigmentation (pers. obs). Furthermore, a small change in chl *a* concentration in response to heat indicates the ability of endosymbionts to tolerate heat stress by just regulating their cell density without significant impact on chl *a* concentration in the remaining viable endosymbiont cells. This response has been found previously for other coral species exposed to thermal stress, leading to 50–80% reduction in their endosymbiont cell densities without any influence on chl *a* concentration [8]. Another study showed changes in endosymbiont density but no apparent effect on chl *a* concentration in *P. acuta* coral exposed to heat [38], suggesting this is a typical response for *P. acuta*. The observed regulation in endosymbiont cell density and chl *a* concentration here translated to minimal changes in F_v_/F_m_ following heat stress exposure, and a subsequent recovery when the stressor was removed. It has previously been suggested that acclimatization mechanism by endosymbiotic dinoflagellates under stressful conditions generally occur by modifying the reaction center (e.g., changing the abundance of the photosynthetic unit such as the Photosystem II) rather than the effective antennae-absorption involving chlorophyll *a* [56]. However, the relationship is very complex due to packaging of pigments and how endosymbiont cells are packed within the coral cells [57]. These results demonstrate *P. acuta*’s thermal tolerance and capacity to recover when conditions are improved. This can be attributed partly to the hosted endosymbionts which are known to be composed mainly of *Durusdinium* (previously *Symbiodinium* clade D) [58,59], a typically stress-tolerant Symbiodiniaceae. Findings here corroborate previous studies on Singapore’s reefs that showed relatively high overall thermal tolerance, particularly corals from Raffles Lighthouse, and identified *Pocillopora* as one of the least susceptible genera [35,60].

While increased thermal tolerance is advantageous for coral survival, elevated temperature is often associated with growth impairment in corals as normal calcification physiology may be compromised [61,62,63,64]. Here, a significant downregulation of *Actin* was observed following heat exposure. Considering this is a major cytoskeletal component involved in growth, downregulation of this gene could be indicative of growth inhibition in response to heat stress. This pattern of *Actin* has previously been observed in similar experiments by Kenkel et al. (2011) [28] and DeSalvo et al. (2008) [65]. We note that *Actin* was initially tested as a control gene in this experiment based on its unresponsiveness to heat in our previous transcriptome-wide study of *P. acuta* [38]. However, the observed active regulation in *Actin* here indicates that its expression pattern may vary widely in this species. The gene’s involvement in coral stress response needs to be further validated. Furthermore, *KIF9*, which is a kinesin-like protein linked to extracellular disassembly also showed changes in expression level following heat exposure. The extracellular calcifying matrix is the site of calcification [66,67], so changes in *KIF9* are likely associated with regulation of the calcification process, which is controlled by exogenous factors such as temperature [68,69,70,71], among others. While these observations are consistent with current understanding of the effects of heat on coral growth and calcification rates, much work is needed to test this hypothesis in the future such as performing concurrent measurements of *Actin* and *KIF9* expression levels and growth indices in corals following exposure to heat stress.

As most corals are sessile, the capacity to constantly sense their environment and respond specifically to a distinct stressor via adaptive immune responses is critical for their survival. Recent research on the genome of *Pocillopora damicornis* (a close relative to *P. acuta*) revealed the occurrence of lineage-specific genes that are associated with the immune response pathway, indicating that this species may have evolved unique immune strategies to cope with changes in the surrounding environment [72]. Here, exposure of corals to heat stress triggered downregulation of *Camk4* expression levels which were then reversed significantly following removal of heat for some specific colonies (G2 and G3), particularly those from Kusu. The pattern of *Camk4* during the recovery period appears to correspond to the site, suggesting a potential influence of natal site on coral responses to heat and its subsequent recovery. Kusu generally has a more variable environment compared to Raffles Lighthouse as it typically experiences strong tidal currents [73] and other external factors such as shipwakes [74] that may facilitate heat dissipation [75]. *Camk4* is a calcium/calmodulin-dependent protein kinase which is associated to adaptive immune response [38] and has been reported to be an upstream regulator of the AMP-activated protein kinase (AMPK) system which is a critical regulator for energy balance observed in mammals [76]. The demand for energy during a stressful condition in corals is high [77], so the regulation of *Camk4* which is activated by presence of Ca^2+^/calmodulin ions [76], is potentially part of *P. acuta*’s acclimatization response. It is interesting to note that the involvement of Ca^2+^ homeostasis disruption associated with thermal stress had been previously reported in cnidarians [65,78,79] which further supports the role of *Camk4* in coral stress response. While it is unclear whether or not corals have an adaptive immune response, the recent report on the genome of *P. damicornis* by Cunning et al. (2018) [72] highlighted some unique immune strategies in corals that have adaptive roles.

Heat-shock proteins [6,80] in corals are also known to alleviate the negative impacts of heat stress by reducing the number of structurally non-native proteins produced by the endosymbionts. In Kenkel et al. (2011) [28], *Hsp16.1* (a small Hsp) was upregulated following heat stress and subsequently downregulated for the recovery period. Conversely, in our study, exposure of *P. acuta* corals to heat showed slight increases of *Hsp16.1*, however, the change was only significant for G2. Interestingly, the expression levels of *Hsp16.1* in heat-stressed corals appeared to increase following the recovery period and this was more pronounced for corals from Kusu compared to Raffles Lighthouse. These changes, however, were not significant. Nevertheless, the observations suggest a likely influence of natal site on the expression pattern of this gene similar to *Camk4* discussed above. While the relationship between *Hsp16.1* level and the water temperature profile of natal sites was not explored in this study, the pattern observed for this gene in response to heat stress corroborates previous observations [20,28,38,81], highlighting its potential as a biomarker. However, considering the further increase of *Hsp16.1* expression even when the stressor was removed, it is unclear what role this gene serves during a heat stress response.

The main findings here indicate strong intercolonial variability in *P. acuta*’s response to heat stress and its capacity to recover. These differences are likely due to genotypic variability between the colonies examined and reflect the potential role this variation has for the resilience of this species in the face of climate change. It is worth mentioning that *P. acuta* corals found in Singapore exhibit variation in branching morphologies: compact and thin [82]. It has previously been shown that the two morphotypes displayed differential responses to thermal stress; the compact colonies were more heat-tolerant than those with thin branches [37]. This difference may be why the colonies examined here—which were of the compact morph—did not display bleaching throughout the experiment, despite the observed changes in photophysiological performances and gene expression levels. However, our findings indicate that even within the same morph, there is substantial variation in stress response. This highlights the importance of elucidating the underlying mechanisms explaining intercolonial variations in this species which may be due to genotypic variability, phenotypic plasticity as reported for other *Pocillopora* species [24,83,84,85], or a combination of both.

Taken together, findings presented here highlight significant regulation of photophysiological attributes and gene expression of *P. acuta* corals in response to changing thermal conditions which, however, depended largely on the coral colony. It is important to note that the underlying factors driving these differences remain unclear and warrant further investigation. In particular, as gene expression is followed by post-transcriptional changes [21,86,87,88], it is critical to examine the expression and turnover of protein products to complete our understanding of the cellular mechanisms behind coral stress response. Future studies should also consider examining specific genes of interest in concert with direct measurements of associated coral-specific phenotypes such as growth and lipid production [89,90] in order to better understand the role of gene regulation in the acclimatization capacity of corals. Such validation studies are an essential step for discovering future biomarkers which can potentially be used as tools for coral restoration management. Nevertheless, our study illustrates that gene expression and photophysiological changes in corals are identifiable, thus paving the way for predictive monitoring of coral stress.

## Figures and Tables

**Table 1:** List of genes of interest (GOIs) and internal control genes (ICGs) (*) with their corresponding functional profile (biological process) and primer designs.

Gene Name (Abbreviation)	Biological Process	Forward Primer Reverse Primer
Actin *	Cytoskeleton	F: 5′-CAA GCA TCC TGT TCT CCT GAC-3′
		R: 5′-AGG TAG GCC GTC AAG TCC C-3′
Acyl-CoA dehydrogenase family member 11 (*ACAD-11*) *	Fatty acid beta-oxidation	F: 5′-TAATCCAGCGACCCAGTGGA-3′
		R: 5′-AAGCCAGGCCTTCTTTTGCT-3′
Heat shock protein 70 (*HSP70*) *	Stress response (heat)	F: 5′-TTTCGACAACAAGGCCACGG-3′
		R: 5′-TCTTCTTCGATCGTTAGGCGG-3′
Calcium/calmodulin-dependent protein kinase type IV (*Camk4*)	Adaptive immune response	F: 5′-GGA ACC CCT GGA TAC TGT GC-3′
		R: 5′-ACA TCG CTT GAT CAC CTC GT-3′
EF-hand domain-containing protein 1 (*EFH1*)	Cell cycle	F: 5′-GTTCTACACCCCAGCTGACT-3′
		R: 5′-CTTGTGGAACAGATGCCACC-3′
Growth arrest-specific protein 8 (*GAS8*)	Cilia motility	F: 5′-TGG AGA AGA AGG AAG CGC AG-3′
		R: 5′-GTA CTC CGA ACG ACT GGA GC-3′
Small heat shock protein 16.1 (*Hsp16.1*)	Stress response (heat)	F: 5′-TGG TCA ACC CTT ACT GCC AT-3′
		R: 5′-TCT CTC TCT GAG CGA TGC TG-3′
Kinesin-like protein (*KIF9*)	Extracellular matrix disassembly	F: 5′-CAA CGG AAC GAT TTT GGC GT-3′
		R: 5′-GAT CCG TAC AGT CAC AGC GT-3′
XIAP-associated factor 1 (*XAF1*)	Apoptosis	F: 5′-TGCGAAAACTGCAACAGACG-3′
		R: 5′-CACAAGGCAAAGACACAGGC-3′

**Table 2:** Results from generalized linear mixed model analysis for changes in endosymbiont density in response to individual factors (treatment, period, site, colony) and interaction between these factors. Significant values are in bold.

Final model = Average Endosymbiont Density~Treatment × Period × Site/Colony			
Fixed effects	Chisq	Df	*p*
Treatment	3.14 × 10^5^	1	**<2.2 × 10^−16^**
Period	2.07 × 10^5^	1	**<2.2 × 10^−16^**
Site	7.33 × 10	1	**0.007**
Treatment × period	1.34 × 10^3^	1	**<2.2 × 10^−16^**
Treatment × site	5.34 × 10^4^	1	**<2.2 × 10^−16^**
Period × site	7.59 × 10^3^	1	**<2.2 × 10^−16^**
Treatment × period × site	3.56 × 10^3^	1	**<2.2 × 10^−16^**
Treatment × period × site × colony	2.58 × 10^6^	16	**<2.2 × 10^−16^**

**Table 3:** Results from linear mixed model analysis for changes in chl *a* concentration in response to individual factors (treatment, period, site, colony) and interaction between these factors. Significant values are in bold.

Final model = Average chl *a* Concentration~Treatment × Period × Site/Colony			
Fixed effects	Chisq	Df	*p*
Treatment	0.46	1	0.498
Period	5.04	1	**0.025**
Site	4.50	1	**0.034**
Treatment × period	0.33	1	0.564
Treatment × site	0.62	1	0.430
Period × site	0.22	1	0.639
Treatment × period × site	3.77	1	0.052
Treatment × period × site × colony	41.57	16	**<2.2 × 10^−16^**

**Table 4:** Results from linear mixed model analysis for changes in Fv/Fm in response to individual factors (treatment, period, site, colony) and interaction between these factors. Significant values are in bold.

Final model = Average F_v_/F_m_~Treatment × Period × Site/Colony			
Fixed effects	Chisq	Df	*p*
Treatment	0.116	1	0.733
Period	95.529	1	**<2.2 × 10^−16^**
Site	1.531	1	0.216
Treatment × period	5.753	1	**0.016**
Treatment × site	0.183	1	0.669
Period × site	0.184	1	0.668
Treatment × period × site	0.659	1	0.417
Treatment × period × site × colony	21.918	16	0.146

## Supplementary Materials

The following are available online at https://www.mdpi.com/2076-2607/8/8/1227/s1, Figure S1: Calculated efficiencies (E) of designed primers for genes of interest—*Camk4*, *Gas8*, *Hsp-16.1*, *KIF9*; and internal control gene Actin, Data S1: Differential gene expression analysis using Poisson-based lognormal model to quantify changes in expression levels of all genes examined across experimental conditions.
