# Supplementary figures and images for: Gene Expression and Photophysiological Changes in Pocillopora acuta Coral Holobiont Following Heat Stress and Recovery
Source: Microorganisms. 2020 Aug 12;8(8):1227. doi: 10.3390/microorganisms8081227 (PMC7463449; doi:10.3390/microorganisms8081227)

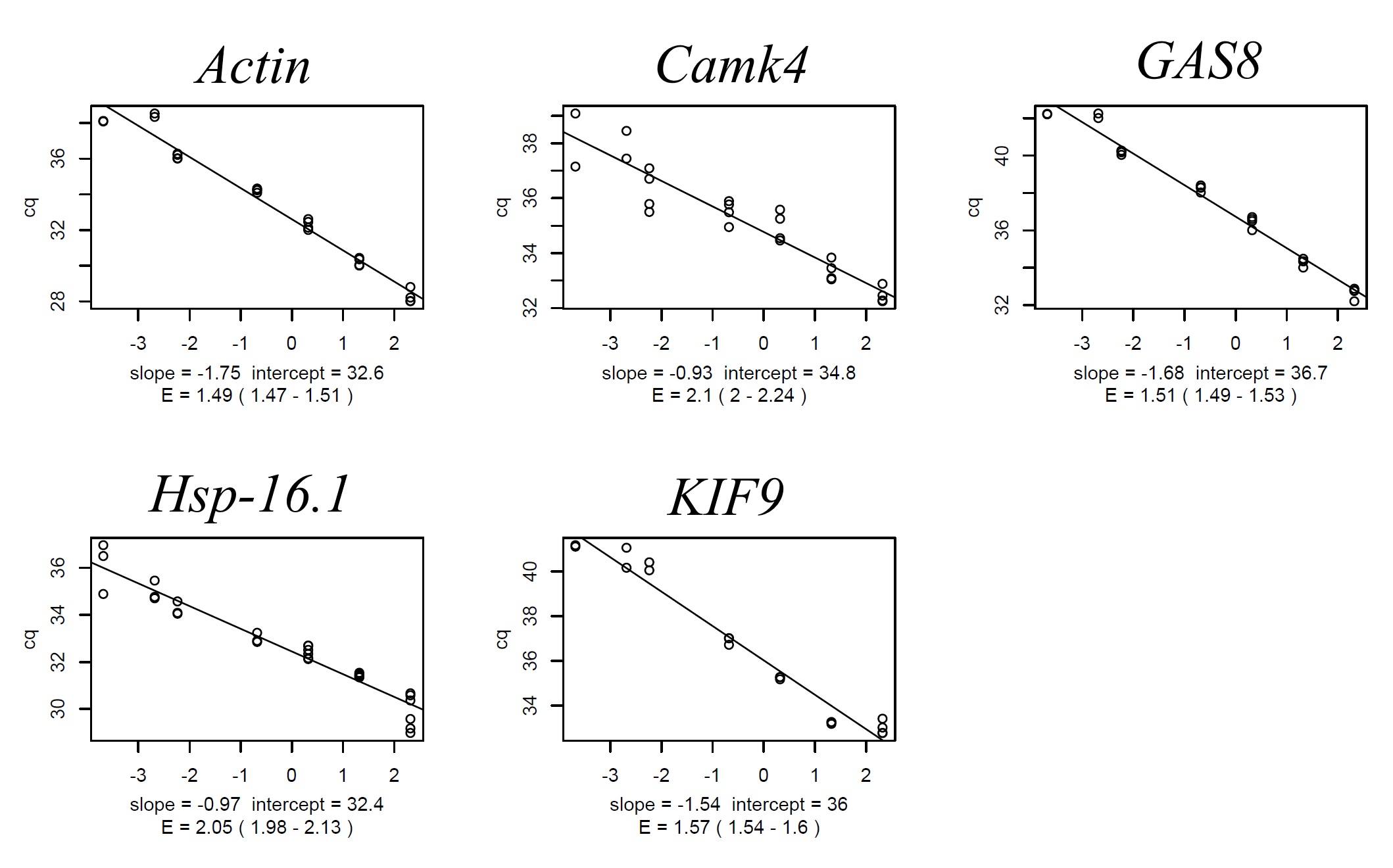

Supplement: Supplementary file 1 [file microorganisms-08-01227-s001.zip › Figure S1.jpg]
